# Trypanotolerance in N’Dama x Boran crosses under natural trypanosome challenge: effect of test-year environment, gender, and breed composition

**DOI:** 10.1186/1471-2156-13-87

**Published:** 2012-10-17

**Authors:** Caleb O Orenge, Leonard Munga, Charles N Kimwele, Steve Kemp, Abraham Korol, John P Gibson, Olivier Hanotte, Morris Soller

**Affiliations:** 1 South Eastern University College (A constituent college of the University of Nairobi), P.O. Box 170–90200, Kitui, Kenya; 2 Kenya Agricultural Research Institute - Trypanosomiasis Research Centre (KARI-TRC), P.O. Box 362, Kikuyu, Kenya; 3 Department of Veterinary Anatomy and Physiology, University of Nairobi, Nairobi, Kenya; 4 School of Biological sciences, University of Liverpool, Liverpool, L69 7ZB, UK; 5 International Livestock Research Institute (ILRI), Nairobi, Kenya; 6 Department of Evolutionary and Environmental Biology, Institute of Evolution, University of Haifa, Haifa, 31905, Israel; 7 The Centre for Genetic Analysis and Applications, University of New England, Armidale, NSW, 235, Australia; 8 School of Biology, University of Nottingham, Nottingham, NG7 2RD, UK; 9 Department of Genetics, The Hebrew University of Jerusalem, Jerusalem, 91904, Israel

**Keywords:** Trypanotolerance, Tsetse challenge, N’Dama, Boran, N’Dama x Boran crosses, Gender effect, Sub-Saharan Africa

## Abstract

**Background:**

Trypanosomosis, a protozoal disease affecting livestock, transmitted by *Glossina* (tsetse) flies is a major constraint to agricultural production in Sub-Saharan Africa. It is accepted that utilization of the native trypanotolerance exhibited in some of the African cattle breeds to improve trypanotolerance of more productive but susceptible breeds, will offer a cost effective and sustainable solution to the problem. The success of this approach is based on the premise that quantitative trait loci previously identified under relatively controlled situations confer useful trypanotolerance under natural field situations. As part of a study to authenticate this hypothesis, a population of 192 cattle, consisting of six batches of N’Dama and Kenya-Boran backcross animals [(N’Dama x Kenya-Boran) x Kenya-Boran] born over the period 2002 to 2006 was constructed. Some of the batches also included pure Kenya-Boran cattle, or N’Dama x Kenya- Boran F1 animals. Each batch was exposed as yearlings to natural field trypanosomosis challenge over a period of about one year; the entire challenge period extending from December 2003 to June 2007. Performance of the animals was evaluated by weekly or biweekly measurements of body weight, packed blood cell volume (PCV), parasitemia score, and number of trypanocide treatments. From these basic data, 49 phenotypes were constructed reflecting dynamics of body weight, packed cell volume (PCV) and parasitemia under challenge.

**Results:**

Females were distinctly more trypanotolerant than males. F1, backcross and pure Kenya- Boran animals ranked in that order with respect to trypanotolerance. Overall batch effects were highly significant (p<0.001) for most traits, and were generally more significant than the gender or genetic type effects. The superior trypanotolerance of the F1 animals was expressed in all three components of animal defense strategies against pathogens: Avoidance resistance, and tolerance.

**Conclusions:**

The results show that trypanotolerance derived from the N’Dama is expressed under field conditions; and that the trait is primarily additive in nature, being expressed in heterozygous condition and in a three-quarters Boran genetic background. The results further, underscore the complexity of the trait in the field manifesting all three host disease-control strategies, and show the importance of gender and local environmental conditions in determining response to challenge.

## Background

About 60 million African cattle [1] are at risk for Trypanosomosis (Nagana), caused by the protozoan parasites: *Trypanosoma congolense, T. vivax, T. brucei *and transmitted by *Glossina* tsetse-fly species. This disease is a constraint to livestock production in Sub-Saharan Africa as effective control methods are not available. However, a degree of resistance to the disease (trypanotolerance) has been reported in some African livestock breeds, among them the N’Dama (ND) longhorn of West Africa [2-6], Orma Boran [7-9] and the Zebu of East Africa [10]. Trypanotolerant quantitative trait loci (QTL) were identified in an F2 ND **×** Kenya-Boran (KB) cattle population under artificial challenge with a single 1180 *T. congolense* clone [11]. This experiment identified 20 trypanotolerant QTLs, eight of which derived their higher trypanotolerance from the Boran, raising the exciting possibility of developing a synthetic breed of higher trypanotolerance than either of the parental breeds (KB or ND). In this context, the backcross (denoted BCB) of the F1 (ND x KB) to the KB is of particular interest, as a potential base population for development of a synthetic breed combining trypanotolerance from the ND and Boran and favourable production traits (primarily body weight) from the Boran; or as a way station for development of a trypanotolerant Boran by marker assisted introgression from the ND. However, in order to translate these results into practical use, it is necessary to ascertain that the QTL responsible for ND trypanotolerance are indeed expressed in a BCB population under field and natural challenge situations with diverse tsetse intensities and trypanosome species and subspecies, coupled with other stressful environmental conditions. We here describe the construction of such a BCB population, and its response to natural field challenge by trypanosomosis under a variety of field conditions.

## Results

### Test-year environment effects (batch effects)

Table 1 shows the results of the fixed effects analysis, including the actual mean trait values of the BC1 males of Batch 1 (n=22) that served as reference group for the fixed effects analysis. Also shown are the estimated mean trait values for each batch, corrected for gender effects; and the maximum significance of the batch effects relative to the reference group; the average values for each trait across all batches (MeanAll); and the within-batch and between-batch coefficients of variation for each trait across all batches.

**Table 1:** Two-Way (batch and gender) ANOVA estimates of batch effects: Ref-mean, mean of BC males of Batch 1 (n=22)

**Trait**	**Ref-mean**^**1**^	**Actual trait mean values by batch**^**2**^						**S**^**3**^	**Mean All**^**4**^	**CV -Batch (%)**^**5**^	**CV-within (%)**^**6**^
		**1**	**2**	**3**	**4**	**5**	**6**				
WIC	60.32	55.57	55.90	57.43	53.37	34.13	43.62		50.00	18.43	51.50
STR	20.05	19.36	18.40	18.18	16.18	8.05	18.58	***	16.46	25.84	72.40
STRTV	15.77	15.43	14.42	13.15	11.48	5.45	15.44	***	12.56	30.24	81.80
STRTC	4.27	3.93	3.98	5.03	4.70	2.60	3.14		3.90	23.49	61.30
TPS	50.05	47.70	51.31	37.25	29.80	16.13	41.85	***	37.34	34.50	93.20
MPAR	2.50	2.46	2.79	2.05	1.84	2.00	2.25	***	2.27	21.62	54.20
%PARD	0.33	0.40	0.31	0.29	0.33	0.15	0.45	***	0.32	31.35	94.10
NINF	5.46	4.56	4.14	4.32	3.98	3.57	4.01	***	4.10	8.23	44.40
NT	5.32	4.36	4.05	4.92	4.53	4.38	4.67		4.49	6.64	27.30
NTI	2.82	1.95	2.22	2.80	2.34	2.23	2.02	**	2.26	13.28	33.30
NIT	0.72	0.74	0.79	0.73	0.77	0.32	0.74	***	0.68	25.90	68.50
MNT	0.09	0.10	0.06	0.07	0.06	0.17	0.10	***	0.09	44.08	119.20
MNT1	0.05	0.03	0.03	0.04	0.03	0.07	0.04	**	0.04	39.82	96.40
DF1	14.27	14.23	15.21	28.86	26.59	24.38	15.40	***	20.78	31.54	70.20
DT1	18.68	23.50	35.41	29.62	68.18	25.34	30.23	***	35.38	46.92	143.20
DC1A	32.96	37.72	51.00	58.79	96.44	51.39	45.17	***	56.75	36.43	113.40
DF2	31.46	37.51	23.73	35.65	57.53	40.13	29.27	***	37.30	31.00	93.10
DCIB	50.14	61.05	55.43	61.12	129.14	66.71	59.65	***	72.18	38.98	112.30
DT2	33.19	42.24	57.32	51.99	33.82	8.01	17.78	***	35.19	54.88	146.40
DC2A	64.52	77.36	79.76	87.79	95.98	47.51	49.63	**	73.00	27.45	68.40
DC12	97.86	113.48	122.16	145.41	195.68	97.73	95.03	***	128.25	29.45	80.10
PCI	31.12	32.34	29.72	36.61	29.55	31.84	30.93	***	31.83	8.14	22.30
MPC	24.10	24.48	24.50	25.01	25.07	22.54	24.22	***	24.30	3.81	10.50
MXPC	33.14	33.41	34.42	35.45	33.45	29.73	35.93	***	33.73	6.56	18.40
MNPC	15.64	16.04	15.48	14.84	16.05	15.49	15.54		15.57	2.87	7.80
PCSR	30.32	30.59	32.51	29.63	29.27	25.51	35.24		30.46	10.76	32.00
PCF1	26.18	26.27	28.33	23.44	29.05	22.58	30.93	***	26.77	12.26	31.20
PCT1	17.46	17.56	16.63	15.79	17.57	18.50	17.30	***	17.22	5.37	15.80
PCF2	25.46	24.74	25.33	25.79	27.01	22.91	25.74	**	25.25	5.41	16.10
PCT2	17.38	17.35	16.40	17.18	18.59	18.17	17.69	*	17.57	4.39	12.50
PCIF1	−4.94	−6.07	−1.39	−13.17	−0.51	−10.46	0.01	***	−5.26	105.94	259.60
PCF1T1	−8.73	−8.70	−11.31	−7.32	−11.65	−3.86	−13.64	***	−9.42	37.44	103.80
PCIT1	−13.66	−14.78	−12.87	−20.78	−11.66	−14.32	−13.63	***	−14.67	21.73	63.00
PCT1F2	8.00	7.33	8.70	10.00	9.25	4.54	8.15	*	8.00	24.05	67.30
WTI	204.32	194.86	196.58	167.93	157.03	150.34	143.66	***	168.40	13.44	35.70
MWT	201.49	188.40	198.00	199.11	167.79	153.95	164.51	***	178.63	10.67	3.00
MXWT	235.82	217.73	230.73	235.31	195.19	176.42	195.99	***	208.56	11.06	3.00
MNWT	171.09	161.86	166.67	161.32	138.94	133.73	130.77	***	148.88	10.82	3.00
WTF1	191.84	181.36	182.72	181.81	150.26	151.08	152.56	***	166.63	10.09	2.00
WTT1	178.09	167.19	175.67	167.48	174.40	135.07	158.70	***	163.08	9.20	3.00
WTF2	181.82	176.73	191.84	176.97	167.09	150.66	146.29	***	168.26	10.28	3.00
WTE	231.19	215.90	201.50	197.16	164.16	140.12	168.33	**	186.92	2.88	22.20
WTIF1	−9.78	−13.60	−11.03	15.04	−1.35	−2.12	15.37	***	0.38	3244.26	258.00
WTIT1	−27.71	−26.88	−25.48	−0.15	6.01	−22.26	9.24	***	−9.92	168.58	37.00
WTC	−2.83	−6.46	1.41	31.19	10.76	3.62	20.86	***	10.23	190.73	42.00
WTC-W	0.03	−0.36	0.41	0.92	−0.24	−3.85	0.85	***	−0.38	469.69	152.00
WTF1T1	−10.82	−11.26	−1.61	−2.93	8.78	−8.89	−2.93	***	−3.14	222.21	64.00
WTT1F2	4.60	−10.73	17.95	28.99	15.33	55.33	11.99	*	19.81	109.73	23.00
WT1E	35.90	31.72	31.76	23.72	24.68	8.24	24.46	***	25.77	11.73	114.42

^1^These animals served as reference group for the fixed effects analysis.

^2^Actual trait mean values by batch, corrected for gender effects.

^3^Mean (All), average trait value across all batches (n=192);

^4^S, maximum significance of an individual batch compared to reference group.

^5^CV (%)-within, mean within batch coefficient of variation combined across all batches;

^6^CV%-batch; coefficient of variation among batch means.

Differences among batches were highly significant for almost all traits, showing the importance of local environment on infection and course of the disease. WIC was distinctly lower for Batch 5 compared to the other batches, and this resulted in lower values for most of the traits that were a function of time (i.e., STR, TPS, and NINF). Batch 5 also experienced a severe drought that compromised the nutritional status and general health of the animals. This resulted in a rapid decrease in PCV under second infection and need for early treatment (DT2=8.01 days). For the same reason, Batch 5 had the lowest proportion of non-treated parasitemia detections (NIT= 0.32) and distinctly lower average weight gain (8.24 kg, Table 1). The season of exposure for Batch 4 was one with a very low tsetse challenge. Consequently, DT1, DC1A, DF2 and DCIB were very high for this Batch. Batches 1, 2 and 6 were high tsetse challenge seasons, taking only 2 weeks until first infection (DF1), while Batches 3, 4 and 5 took 3–4 weeks. Batches 1, 2 and 6 also presented higher MPAR, indicating that under high challenge not only does infection occur more rapidly, but parasitemia load is also greater. The animals in Batches 1 and 2 were the first animals in the experiment and were allowed more time in acclimatizing before exposure to tsetse. Consequently, the animals in these batches were slightly older (by a few months) than in the other batches and their WT1 was correspondingly greater. They also experienced the highest average weight gain of 32 kg from the initial WTI to final weight in the study period.

Coefficients of variation (CVs) were generally considerably higher within batches (median, 40-50%) than between batches (median, 10-20%). This is expected, since the averaging effect of many individuals in each batch will tend to level variation among batches relative to variation within batches. CVs were quite high for the parasitemia, infection and treatment traits (50-90% within-batches; 10-50% between-batches). This is plausible, considering the multiple intrinsic, environmental and chance factors that affect these traits. CVs were considerably lower for the PCV and WT traits (<30% within-batches; <10% between-batches), implying that these are more directly determined by intrinsic animal factors. Exceptions to this were the traits involving differences or changes (PC1F1, WT1F1, WT1T1, WTC, WTC-W, WT1F1T1 and WTT1F2). For such traits, the MeanAll value (the denominator of CV) is low, since the change for the individual animal can be positive or negative; while standard deviation (the numerator of CV) is large. The net effect of a small denominator and large numerator is a very high CV. Thus, for these traits CVs are not meaningful.

### The course of infection and treatment in an “average” BCB animal

Based on the gender-corrected MeanAll trait values across all batches (Table 1), the average BCB individual was infected 21 days after exposure (DF1) and required treatment 35 days after infection (DT1). The individual became re-infected 37 days after treatment (DF2), and was treated again after 35 days (DT2). Thus, there was a distinct increase in days to infection but no difference in days from infection to treatment for second infection as compared to first infection. On average, the individual underwent 4.10 infection cycles (NINF) in the course of 50 weeks of exposure (WIC), requiring 4.49 treatments (NT). Out of these, about half (50%) required treatment when they reached the critical 18% PCV (NT1), while the remaining treatments were required at a PCV above the 18% threshold. Positive parasitemic cases (STR) were detected on average in 16.46 tests across the entire challenge period of 50 weeks. Thus, on average, 68% of parasitemic detections did not result in treatment (NIT). Out of the total parasitemic detections (excluding mixed parasitemic infections), 76% were due to *T. vivax* (STRTV) and 24% were due to *T. congolense* (STRTC). Hence *T. vivax* was the primary pathogen. This is consistent with previous studies [12] that also implicated this parasite as the main pathogen in these humid and sub-humid tsetse infested regions of Africa. The mean parasitemic score for parasitemic detections (MPAR) was 2.27, equivalent to 10^2^-10^3^ trypanosome parasites per μl of blood per positive sample.

On average, PCV decreased by 9.42% (absolute decrease) from first infection to treatment (PCF1T1) at a rate of 0.27%/day and recovered (increased) by 8.00% after treatment at a rate of 0.22% per day. Thus, the lost PCV due to infection was not completely recovered after treatment of first infection to the start of second infection cycle. On average, across the entire challenge period, mean PCV (MPC) was 7.5% less (absolute value) than at the beginning of the experiment (PCI), the loss being due to recurrent infection. Thus, for the entire year the animals were functioning at only 75% of normal PCV values.

On average, animals lost 3.14kg (WTF1T1) during the first infection cycle at a rate of 0.088 kg per day (WTF1T1/DT1); and gained 19.81 kg (WTT1F2) before re-infection from the end of the first cycle to beginning of the second cycle, at a rate of 0.56 kg/day (WTT1F2/DT2). Since the rate of weight gain after treatment was higher than rate of weight loss, the animals gained net weight. Thus, treatment had strong positive effect in regaining lost PCV and allowing further increase in body weight during challenge.

Average weight gain by batches across the challenge period (Table 2) ranged from 23.72 to 31.72 kg, with mean of 29.28 kg, not including Batch 5, where gain was very low (8.24 kg) due to poor nutritional conditions as discussed above. Thus, despite infections, the animals gained weight. This is probably due to the fact that the animals were about a year old at start of challenge period and hence still very much in their growth phase. Standard deviation of weight gain, however, was 29.49 kg, almost exactly equal to the mean. Thus, while most animals gained weight, an appreciable fraction (about 15%) lost weight. This may be a reflection of segregation of trypanotolerance loci in the BCB population. Comparing genetic types, there was a strong interaction with batch conditions. Thus, in Batch 4, which had very low tsetse challenge and good environmental conditions, the KB gained more than the BCB. But in Batch 5, which was a severe drought year, BCB gained more than KB. Similarly, in Batch 6, which was a high challenge year, the F1 gained more than the BCB; although in the absence of challenge, we would expect breeding value for weight gain of the BCB to be greater than for the F1.

**Table 2:** **Average overall weight gain (WG) by batch**^**1**^

**Batch**	**Type**^**2**^	**No.**^**3**^	**WG (kg)**^**4**^	**SD**^**5**^	**CV (%)**^**6**^
1	BCB	39	31.72	29.07	91.67
2	BCB	34	31.76	29.23	92.03
3	BCB	32	23.72	29.21	123.14
4	BCB	19	24.68	27.33	110.73
	KB	13	36.58	29.99	82.01
5	BCB	17	8.24	22.95	278.68
	KB	10	4.20	12.02	286.11
6	BCB	39	24.46	32.02	130.92
	F1	35	39.82	29.66	74.47

^1^The average overall weight and coefficient of variation is for BC, KB and F1 cattle from initial weight (WTI) to the final weight in the last one month of study.

^2^Type, genetic type.

^3^No., number of animals;

^4^WG, weight gain;

^5^SD, standard deviation;

^6^CV, coefficient of variation (%).

### Female gender effect in relation to trypanotolerance

Table 3 shows a more elaborate analysis for gender effects (female trait value as a deviation from the corresponding male trait value) and their significance, than our previous study [12]. Also shown is whether the direction of effect is in the direction of greater tolerance (R) or greater susceptibility (S). Statistically, the comparison is strongest for the BCB as it has the largest sample size (88 males, 104 females). Across all three genetic types, 10 tests did not reach significance while 9 tests were significant. Of the significant tests, 7 were highly significant, (p<0.001), out of which 6 (86%) had the female as the more resistant gender and only 1 test (14%) indicated that the male was the more tolerant gender. Two tests were significant (p<0.01) and in both of these, the female was the more tolerant gender. Thus overall, 89% of female effects, that were at least significant, were in the direction of higher resistance. For the most part, the traits for which the female presented the more susceptible phenotype were scattered apparently randomly among the trait groups. The same holds true for the phenotypes for which the greater resistance of the female was statistically significant. Also, the proportion of resistant female phenotypes was highest and significance was greatest for the large BCB sample as compared to the smaller F1 and KB samples. These observations are consistent with the females being more resistant across all traits, with the scattered instances where the female presents the more susceptible phenotype simply representing sampling variation from a basically resistant population.

**Table 3:** Gender effects, female effects as deviation from the male

**Trait**	**BCB (88/104)**^**1**^		**F1 (24/13)**^**1**^		**KB (7/8)**^**1**^	
	**Effect**	**R/S**^**2**^	**Effect**	**R/S**^**2**^	**Effect**	**R/S**^**2**^
WIC	1.69		9.82		−12.80	
%PARD	−0.009	R	−16.43	R***	5.00	S
STR	−0.756	R	−1.54	R	−5.80	R
STRTV	−0.205	R	−1.08	R	−3.10	R
STRTC	−0.551	R	−0.46	R	−2.70	R
TPS	−1.673	R	−4.11	R	−9.90	R
MPAR	0.105	S	−0.14	R	−0.02	R
NINF	−0.826	R***	0.05	S	−1.11	R
NT	−0.899	R***	0.21	S	−0.67	R
NTI	−0.333	R*	−0.31	R	−1.16	R**
NIT	0.056	R*	−0.07	S	0.12	R
MNT	−0.009	R	−0.014	R	−0.02	R
MNT1	0.003	S	−0.0197	R	−0.02	R
DF1	3.343	R	−9.01	S	40.01	R*
DT1	7.75	R	12.92	R*	−31.02	S
DC1A	11.607	R	3.93	R	8.02	R
DF2	10.663	R***	1.41	R	−9.70	S
DCIB	16.255	R*	14.32	R*	−41.01	S
DT2	27.934	R	5.13	R	−32.02	S
DC2A	35.965	R***	6.51	R	−42.03	S
DC12	43.926	R***	10.42	R	−34.02	S
PCI	1.546	R	−0.43	S	1.11	R
MPC	0.904	R	1.09	R	0.89	R
MXPC	1.523	R	1.55	R	0.71	R
MNPC	−0.129	R	0.38	R	1.38	R*
PCSR	1.667	R	2.79	R**	1.19	R
PCF1	1.278	R	−3.21	S*	−2.31	S
PCT1	0.095	R	0.57	R	−0.11	S
PCF2	1.074	R*	2.23	R	−1.12	S
PCT2	−0.38	S	−0.52	S	2.83	R*
PCIF1	−0.535	S	−2.75	S	−3.90	S*
PCF1T1	−1.47	S***	3.77	R*	2.19	R
PCIT1	−1.657	S*	0.02	R	−1.04	S
PCT1F2	0.991	R	1.36	R	−0.71	S
MWT	−6.788		−0.12		3.61	

***, P<0.01; **, P<0.05; *, P<0.10.

^1^In parentheses, (M/F) M, number of males and F, number of females on which the estimates of effects are based; ^2^Column R/S: R, female shows the more resistant trait value; S, female shows the more susceptible trait value.

Female resistance was unequivocally expressed in all three genetic types in most of the phenotypes constructed to reflect various aspects of trypanotolerance: Females took longer (3 and 40 days in BCB and KB, respectively) to become infected after first exposure (DF1), 7 and 13 days in BCB and F1 respectively to require treatment after infection (DT1), 11 days (p<0.001) and 1 day ( in BCB and F1, respectively ) to become reinfected (DF2), and 28 days (in BCB) and 5 days (in F1) to require treatment after re-infection (DT2). Total number of infections (NINF) and treatments (NT) were less (p<0.001 in BCB females), and all genetic type females maintained generally higher PCV values (MPC, p<0.01 in KB females) across the challenge period. Phenotypes reflecting changes in PCV as a result of infection or treatment were more mixed in direction. However, these are difficult to interpret. As noted above, a larger decrease in PCV from infection to treatment may reflect greater sensitivity, but also may simply reflect the fact that the more tolerant animal starts with a higher average PCV, while PCV at treatment is fairly constant at 18%. Interestingly, weighted average body weight of the females across all three genetic types, was only 5.4 kg less than for males. The difference is smaller than found between males and females at two years of age, in locations not subject to trypanosomosis challenge. This too, may reflect better ability of the females to cope with trypanosomosis challenge compared to males, under field conditions.

Table 4 shows effects and their significance and direction, for the comparison of genetic types. The F_1_ was distinctly more tolerant than the BCB, showing the more resistant phenotype in 22 out of the 33 traits tested. The difference is even more striking when considering non-significant and significant effects; 15 of the F_1_ resistant effects were in this category (8 at p<0.01), while only 4 of the effects indicating greater susceptibility were significant or not significant. The effect of ND genome is less apparent in the comparison of KB to BCB. The BCB showed the more resistant phenotype in just 17 out of the 33 traits. However, the difference is much more apparent when considering non-significant or significant effects. Ten of the resistant effects were in this group for the BCB and only one for the KB. The resistance conferred by the ND genome is dramatically expressed in the F_1_ to KB comparison. For 28 of the 33 traits the F_1_ displayed the more resistant phenotype. In 18 instances the effect was not significant or significant (7 at p<0.01). None of the traits for which the KB was the more resistant reached significant levels. From the results it is clear that ND tolerance is displayed strongly in heterozygous state, but more strongly in the F1 to BCB comparison than in the BCB to KB comparison. This aspect of the results will be treated more extensively in the Discussion section.

**Table 4:** Genetic type effects

**Trait**	**F1 – BCB**^**1**^**(39/37)**^**4**^		**KB-BCB**^**2**^**(23/32)**^**4**^		**F1-KB**^**3**^**(39/23)**^**4**^	
	**Effect**	**R/S**^**5**^	**Effect**	**R/S**^**5**^	**Effect**	**R/S**^**5**^
WIC	−4.81		−9.95		5.15	
%PARD	−17.20	R***	2.05	R	−19.25	R***
STR	−8.23	R***	−0.75	S	−7.48	R**
STRTV	−6.29	R***	−1.82	S	−4.49	R**
STRTC	−1.84	R***	0.33	S	−2.17	S
TPS	−16.91	R***	−2.15	S	−14.75	R**
MPAR	0.132	S	−0.02	S	0.152	S
NINF	−1.12	R***	−0.26	S	−0.86	R*
NT	−0.95	R**	−0.05	S	−0.91	R*
NT1	−0.372	R*	−0.28	S	−0.09	R
NIT	−0.062	S*	−0.39	S***	0.328	R***
MNT	−0.0195	R	−0.02	S	0.0005	R
MNTI	−0.0119	R	−0.02	S	0.0081	R
DF1	15.01	R***	−5.36	R**	20.36	R**
DT1	−10.03	S**	−25.41	R	15.41	R
DC1A	4.91	R	−30.77	R**	35.67	R**
DF2	1.32	R	−13.21	R	14.51	R*
DCIB	−8.72	S	−38.62	R*	29.92	R*
DT2	−1.61	S	14.41	S	−16.01	R
DC2A	−1.83	S	1.22	S	−3.01	R
DC12	3.01	R	−29.58	R*	32.58	R*
PCI	2.65	R**	0.93	S	1.72	R*
MPC	3.87	R***	−1.35	R***	5.22	R***
MXPC	2.81	R**	−2.26	R***	5.06	R***
MNPC	0.33	R	−1.05	R*	1.38	R*
PCSR	2.63	R**	−2.95	R***	5.58	R***
PCF1	−2.15	S*	−2.29	R	0.14	R
PCT1	−0.85	S	−0.23	R	−0.62	R
PCF2	2.86	R**	−1.71	R***	4.57	R***
PCT2	−0.41	S	−1.19	R	0.79	R
PCIF1	−4.83	S**	−3.25	R	−1.55	S
PCF1T1	1.44	R	1.62	S	−0.18	S
PCIT1	−3.14	S**	−1.22	S	−1.92	S
PCT1F2	2.63	R**	−2.05	R***	4.68	R***
MWT	−28.21	E***	15.74	E***	−43.94	E***

^1^F1-BCB, F1 effects as deviation from BCB, by one-way ANOVA of data from Batch 6.

^2^KB-BC, KB effects as deviation from BCB, from combined one-way ANOVA of data from Batches 4 and 5; ^3^F1-KB, F1 effects as deviation from KB; ^4^In parentheses (F1, KB/BCB) number of F1, KB and BCB animals in each estimate; ^5^Columns R/S, R indicates that the genetic type with higher proportion of N’Dama genome is the more resistant; S indicates the reverse. E, effect is in the expected direction.

## Discussion

To the best of our knowledge, this is the first exhaustive phenotyping study where a cattle population has been constructed by a defined cross between trypanotolerant and susceptible breeds, and evaluated under natural tsetse- and trypanosomosis challenge. Phenotypes measured included number of infections, treatments, body weight, PCV and parasitaemia scores. The animals were allowed to graze in natural pastures and habitats infested with different species of tsetse flies transmitting different species and subspecies of trypanosomes, of varying virulence and intensities. This gave an opportunity to assess the actual behavior of the experimental animals in real life conditions in the presence of interaction of the environment, disease (trypanosomosis) and the vector (tsetse fly). To a large extent it followed the practices of an average, modern African livestock farmer and therefore, the results presented may be assumed to reflect the situation on the ground.

### Test-year environment effects analysis

Overall, test-year environment (batch) effects were highly significant (p<0.001) for most traits, and were generally more significant than the gender and genetic type effects. Very high tsetse fly and trypanosome challenges were experienced for Batches 1, 2 and 6. Batches 4 and 5 experienced low tsetse fly challenge interspersed with severe drought. The above two varying conditions clearly reflected the effect of tsetse challenge and trypanosomes on one hand and food deprivation on the other. For example, under high tsetse challenge (Batches 1, 2 and 6), animals on average took only two weeks to be infected (DF1), while under low tsetse challenge (Batches 4 and 5) animals took 4 weeks to be infected. Yet, animals in Batches 4 and 5 recorded the lowest PCV parameters across all batches, showing the effect of poor nutrition on PCV. This shows why treatment should be based on both screening for parasites and PCV determination and not on PCV alone. The batches that experienced low tsetse challenge but severe drought (Batches 4 and 5) gained more weight after treatment and before re-infection (WTT1F2) than those batches that experienced high tsetse challenge. Thus, in times of drought it may be prudent to treat undernourished but ‘uninfected’ animals prophylactically for them to achieve better weight gains.

### Gender and genetic type effects

Overall, the results of this study identify the female gender, and the genetic types with higher proportion of ND genome (i.e., F1 vs. BCB, BCB vs. KB, and F1 vs. KB), as the more trypanotolerant types. Within each specific comparison (e.g., female vs. male comparison in BCB, F1 and KB; or higher vs. lesser proportion of ND genome in F1 to BCB, BCB to KB, or F1 to KB comparison), there are some traits for which the supposedly less trypanotolerant type manifests the more tolerant phenotype. Similarly, within each specific gender or genetic type comparison, different traits show different levels of significance (non-significant, significant or highly significant). Examination of Tables 3 and 4 shows that degrees of significance and anomalous trait directions appear to distribute more or less randomly among the specific comparisons, and are not concentrated at specific traits. We believe that the most plausible explanation for these observations is that they represent sampling variation from a more trypanotolerant population value. That is, we propose that on a population basis, the trypanotolerant types (female vs. male, F1 vs. BCB or KB, BCB vs. KB) are more trypanotolerant across all of the traits, and variation in observed direction and degree among specific traits and comparisons are a matter of sampling variation only. With so many traits, types and comparisons, sampling variation in the less tolerant direction from a more tolerant population coupled with the converse from the less tolerant population, can give a contrast for which the more tolerant type have the less tolerant phenotype. Sampling in the opposite direction can yield a highly significant difference in the expected direction in one trait, non-significant difference in the expected direction for some other trait. Thus, we do not attribute variation in direction and degree of effect among the difference specific comparisons as representing true population differences in these attributes, and hence we will not discuss these differences in detail.

Combining all observations, then, we find that greater trypanotolerance whether conveyed by female gender or ND genome is associated with a lower number of positive trypanosome detections over the entire challenge period (STR); fewer infection cycles (NINF) and fewer treatments (NT); more days until first and second infection after exposure (DF1 and DF2), and from date of infection to treatment (DT1 and DT2); and higher average PCV across the entire infection period (MPC). Thus, this experiment fully achieved one of its primary goals in showing that trypanotolerance is expressed across the entire range of a challenge period, and in all aspects of trypanotolerance.

The finding of greater trypanotolerance of the female gender is in agreement with previous work reported [12,13], the latter in Galana ranch involving Orma Boran cattle where in both cases, it was shown that females required fewer treatments than their male counterparts. Also, male vs. female weight-gain difference across the challenge period was less than the difference between male and female when reared for a comparable period in the absence of trypanosomiasis challenge.

Equally, this finding that female gender may be more trypanotolerant than the male is an important one as it confirms a similar hypothesis arising from a comparative study on susceptibility of male and female albino mice to Trypanosome brucei brucei, whose results suggested that the female mice were more trypanotolerant than their male counterparts, due to the fact that the polyclonal β-Lymphocyte factor is more active against trypanosomes in females than in males [14]. It has also been reported that male N’Dama have higher plasma cholesterol levels than females and susceptible cattle have higher levels of plasma cholesterol and triglycerides than trypanotolerant cattle [15]. Reasons for gender differences between levels of plasma cholesterol and triglycerides in both breeds are not known. However, it has been postulated that the low plasma lipid values of N’Dama would suggest that this animal has limited lipid nutrients to support the growth of trypanosomes, hence development of lower parasites during infection [16]. Trypanosomes take up cholesterol, phospholipids and total lipids for growth. They also serve as sources of energy for trypanosome metabolism. These postulations may also explain the differential expression of trypanotolerance in the different genders but more exhaustive studies are needed to confirm this. Other possible explanation for the effect of gender on trypanotolerance points to the fact that trypanotolerant QTLs may be sex-linked or they may be in linkage disequilibrium with sex genes.

All in all, keeping female animals may be an attractive option in tsetse endemic areas, particularly when farmers are encouraged to put more emphasis on milk production and less use of draft power by bulls for field cultivation.

With respect to effect of ND genome, the F1 was clearly superior to the KB in all aspects of trypanotolerance, with minor exceptions. Since all trypanotolerance loci in the F1 are present in heterozygous state, this means that to a large extent, gene action at the trypanotolerance loci must have a strong additive component, in addition to any dominance or recessive effects. This conclusion differs from that based on QTL mapping in an F2 population [13], which reported primarily recessive gene action at their mapped trypanotolerance loci. Those conclusions [13] were based on rather complex QTL mapping analyses, while the F1 to BCB and BCB to KB results in the present study, although based on a smaller sample, are strong and direct. Therefore, we believe that they should be taken as representing the actual reality. This means that QTL mapping for trypanotolerance based on the BCB should be effective. If trypanotolerance loci were indeed recessive, they would not come to expression in the F1 or BCB.

The apparent superiority of the F1 in trypanotolerance relative to the BCB was greater than the apparent superiority of the BCB relative to the KB. The F1 was superior in 22 of 33 traits, of which 16 were at the significant and highly significant levels; while the BCB was superior to the KB in only 17 of 33 traits of which 7 were at the significant and highly significant levels. Yet on an individual locus basis, the expected additive superiority of the BCB to the KB is the same as the expected superiority of the F1 to the BCB. In the F1, all trypanotolerance loci are present in all individuals in heterozygous state; in the BC1 sample, half of trypanotolerance loci are present in all individuals in heterozygous state; in the KB, none of the trypanotolerance loci are present. Thus, across all loci, the additive population value for trypanotolerance of the BCB is as far removed from the population value of the KB, as the population value of the F1 is removed from the population value of the BCB. Similar unexpected results were obtained for mean body weight. The F1 was distinctly lighter than the KB (−43.94 kg), indicating an appreciable additive component for gene effect of body weight loci from the KB. But here too, the difference between F1 and BCB (−28.2 kg) was almost twice as great as the difference between BCB and KB (−15.74 kg). The explanation for these anomalies may lie in the specific environment of Batches 4, 5 and 6. Batches 4 and 5, on which the BCB vs. KB comparison is based were characterized by low tsetse challenge, and poor nutrition due to drought. This would have reduced differences due to trypanotolerance and growth rate. In contrast, Batch 6 on which the F1 to BCB comparison is based was characterized by high tsetse challenge and good nutrition. This would have allowed fuller expression of differences in trypanotolerance and growth rate.

In summary, the BCB individuals demonstrated increased ability to manage trypanosomosis as compared to purebred KB and were intermediate to a sample of F1 individuals. This shows a strong additive component to trypanotolerance loci, validating the use of the BCB as a mapping population (the results of the QTL mapping component of the study will be reported separately), and as a platform for development of a synthetic breed combining trypanotolerance of the ND with body size of the Boran. In addition to the comparison of genetic types, the results demonstrate clearly superior trypanotolerance of the female gender as compared to the male within all three genetic types (KB, BCB and F1), again supporting our previous study [12] even more as in the present case many more traits were analyzed. Thus, female BCB animals may be superior cow-mothers under field challenge. The study was unique in following a population under challenge over the course of time, showed that trypanotolerance was also reflected in the time course of infection, treatment, recovery and re-infection.

### Trypanotolerance and the three components of animal disease resistance

The defense strategy of a host animal with respect to disease resistance has three components: avoidance, which aims to reduce the risk of exposure or infection; resistance, mediated primarily by the immune system, which aims to reduce pathogen burden following infection; and tolerance, which aims to reduce the deleterious effects of pathogen burden on the host [17]. In a striking study [18] based on construction of Boran/N’Dama chimeric twins, it was shown that N’Dama trypanotolerance included both resistance, expressed as ability to control parasitaemia; and tolerance, expressed as ability to control anemia. Moreover, control of parasitemia and control of anemia were shown to be separate traits. All three defense strategies are exemplified in the F1 to KB comparisons of the present study and also in our previous study [12]. The F1 animals had better ability than KB to control parasitemia, as shown by a lower percentage of positive parasitemia scores (%PAR) and lower total parasitemia score (TPS), exemplifying resistance. They had better ability than the KB to control the effects of pathogen burden, as shown by higher mean PCV across the entire test period (MPC), higher percentage of parasitemia detections that did not require treatment (NT1), longer interval from first parasitemic detection to treatment (T1), and fewer trypanocide treatments across the challenge period (NT), exemplifying tolerance. Finally, as uniquely shown in the present study, the F1 had better ability to avoid infection, as shown by delayed onset of first infection (DF1), and greater elapsed time between end of first infection and onset of second infection (DF2), exemplifying avoidance.

## Conclusions

This study shows that trypanotolerance observed under highly controlled conditions is also effective under field challenge. Also, females were distinctly more trypanotolerant than males. F1, backcross and Kenya Boran animals ranked in that order with respect to trypanotolerance, showing clear co- or partial dominance of trypanotolerance loci. Overall batch effects were highly significant (p<0.001) for most traits, and were generally more significant than the gender or genetic type effects, showing the importance of local environmental conditions in determining response to challenge. The superior trypanotolerance of the F1 animals was expressed in all three components of animal defense strategies against infection by pathogens: Avoidance, resistance and tolerance.

## Methods

### Ethical approval

ILRI’s Institutional Animal Care and Use Committee (IACUC) is mandated to review all experimental procedures and experiments on animals within ILRI’s watch and approve or disapprove. After the committee considered ethical issues and physical procedures associated with the housing, feeding, experimentation, and all other routine matters pertaining to the normal welfare of the animals, it approved the experimental research reported here through written permission.

### Study site

**Figure 1:**
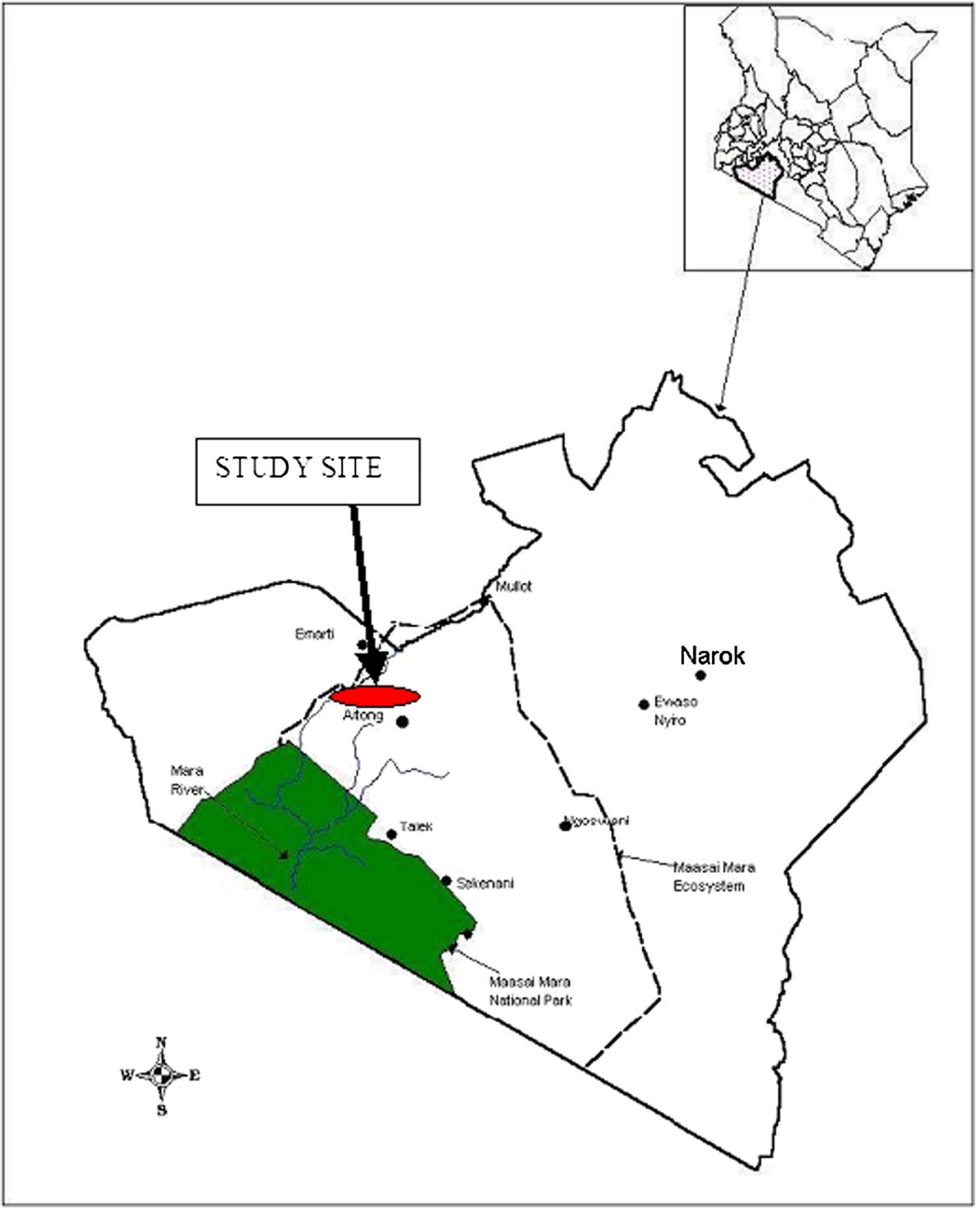
A map of Kenya showing Narok field study site (the green area shows the mara-serengeti ecosystem).

The field study site was located in Narok District, Southwest Kenya between 10.00’S and 10005’S, and 35005'E and 35015’E (Figure 1). The altitude of the area ranges from 1600 to 2130 m above sea level. The study area is classified as semi-arid, with annual rainfall of 750–1250 mm. The rain comes in two seasons: the ‘long’ (main) rains in March to May and the ‘short’ rains in November to December, with two dry periods in January to February and June to October. The study area is primarily savannah grassland, but has four distinct types of vegetation patterns: open grassland, wooded grassland, dense thickets and African acacia genus. There are three tsetse species in the study area: *Glossina swynnertoni, G. pallidipes, G. fuscepleuris,* but the first two species are predominant. The site is typical of the tsetse infested arid and semi-arid areas of Kenya and was selected on the basis of previous observations of high trypanosomosis prevalence in cattle during field surveys by the Kenya Trypanosomiasis Research Institute (KETRI, Kikuyu, Kenya). The presence of abundant wildlife is an important feature of the study area, being part of the 25,000 sq. km rangelands forming the Mara-Serengeti ecosystem.

### Breeding and management of the backcross population

In the course of the Hanotte *et al*. [11] QTL mapping study, a frozen bank of semen from 13 F1 (ND x KB) males was established at the International Livestock Research Institute (ILRI, Nairobi, Kenya). The population from which these F1 sires were derived was constructed by single pair matings of 4 ND males (ND7, ND8, ND9 and ND10) and 4 KB females (KB1419, KB1688, KB1801 and KB2094) resulting in 13 F1 (ND **×** KB**)** males (henceforth: F1 sires) belonging to four full-sib families according to their ND sire and KB dam. In the present study, semen of these 13 F1 males was used to produce 192 backcross (BCB) animals through insemination of about 200 KB females at the ILRI Kapiti ranch (a tsetse free zone), over the period of June 2001 to December 2006. Doses of semen from individual sires were used in rotation so that each successive dose of semen was from a different sire, in order to maximise equality of sire representation over time. As the matings proceeded and information on successful pregnancies became available, semen usage patterns were adjusted to maintain, as much as possible, equal representation of sires among live-born progeny. At the final count, the four F1 sire-families were represented by 42, 33, 65 and 52 BCB progeny, respectively (total: 88 males and 104 females, Table 5).

**Table 5:** Batch composition by sex and genetic type and duration of tsetse challenge period

**Batch**	**Type**	**Male**	**Female**	**Total**	**Start (m.yr)**	**Days**
1	BCB	22	22	44	12.03	389
2	BCB	14	22	36	02.04	391
3	BCB	14	18	32	10.04	402
4	BCB	9	13	22	04.05	374
	KB	7	8	15		
5	BCB	10	9	19	08.05	239
	KB	8	0	8		
6	BCB	19	20	39	07.06	305
	F1	24	13	37		
Total	BCB	88	104	192		
	KB	15	8	23		

Type, genetic type: KB, Kenya Boran; F1, F1 product of N’Dama male x KB female; BCB, Backcross product of F1 male and KB female. Start, month and year of entry to tsetse challenge; Days, total days on challenge.

In creating the animal population for this experiment, artificial insemination was used. Estrus detection was by experienced herdsmen who in turn reported to the inseminators. Due to cost, estrus synchronization was not implemented. However, possibly due to inadequacies in heat detections, numerous repeat inseminations were required before some of the cows conceived, leading to overall low conception rates. Consequently, the full planned numbers of experimental animals could not be achieved in a single season. This in turn led to exposing the animals to tsetse and trypanosomosis challenge in the field in batches, which encountered different weather conditions and therefore different tsetse fly intensities and different nutritional conditions. This enabled effect of variation in environment to be evaluated by calculation of Batch effects.

Purebred KB calves born contemporaneously with the BCB calves at Kapiti were included in Batches 4 and 5. In addition, frozen semen from two ND bulls (ND164 and ND162) was used to inseminate purebred KB dams at Kapiti to produce F1 calves, born contemporaneously with the BCB calves of Batch 6. ND164 produced 29 F1s while ND162 produced 10 F1s to make the total of 39 F1s. The KB and F1 calves were reared alongside their contemporaneous BCB calves and under the same management regimes throughout the experiment.

Each calf born in Kapiti was weighed at birth, and subsequently at monthly intervals until transported to the field site. Each calf was assigned a unique ear tag, a tattoo on the ear and a brand burned on the rump that uniquely identified the animal and was associated with pedigree information on the animal.

Progeny were run as suckler calves with their dams on natural pasture without feed supplementation. In accordance with standard ranch management conditions, all cattle were treated with acaricides (Triatix®) every two weeks to control ticks; vaccinated quarterly against Foot and Mouth Disease (FMD); and dewormed both prophylactically and tactically (e.g. in anticipation of a worm outbreak following the rains) until weaning at 8 to 10 months of age. Male calves were not castrated. Weaner groups were transported to the KETRI Muguga ranch before being taken to the KETRI operated challenge pastures at Narok ranch. In Muguga, they were again vaccinated against FMD and also against Contagious Bovine Pleuropneumonia (CBPP) and East Coast Fever (ECF), diseases that are known to be endemic at the Narok ranch, The BCB progeny were moved to the Narok field site in six different batches, with start dates between December 2003 and July 2006, and end dates between March 2005 and June 2007 (Table 5). There was considerable overlap between field challenge dates of the different batches. Batches 1, 2, 3 overlapped one another to some extent, as did Batches 3, 4, 5. There was a one-month overlap between Batches 5 and 6. Each Batch was exposed to field tsetse challenge over the course of a year or more, with the exception of Batch 5 that was kept under field challenge conditions for 239 d.

The genetic-type comparison study included 71 BCB cattle (38 males and 33 females) in Batches 4, 5 and 6; 23 purebred KB (15 males and 8 females) in Batches 4 and 5; and 37 F1 (13 males and 24 females) in Batch 6.

### Definition of phenotypes and traits

From the raw phenotypes recorded weekly or biweekly for each individual (PCV, PAR, BW and NT) various “traits” were meticulously defined and constructed making a total of 49 phenotypic traits (Table 6). Many of the traits included a “time” component, e.g., DF1, days from first exposure to infection. Traits were classified into groups as absolute body weight traits (BW), PCV traits (PC), parasitemia traits (PT), infection cycle traits (IC), treatment-related traits (TT), body-weight-change traits (BWC). Traits thought to be associated with trypanotolerance were classified with respect to direction of effect, i.e., as to whether high trait-value indicated a higher degree of trypanotolerance (H-traits) or low trait-value indicated higher trypanotolerance (L-traits). For example, DF1, days to first infection after transfer to Narok, was classified as an H-trait since a larger number of days to first infection was an indication that the animal was more trypanotolerant than one that became infected after a shorter period. In contrast, MPAR, mean parasitemic score, was classified as an L-trait, since a low mean parasitemic score indicated that the animal was better able to control the parasitemia as compared to an animal with a higher mean parasitemic score; thus indicating higher trypanotolerance. Where neither of these applied, the trait was listed as not relevant to trypanotolerance (NR). NR-traits included: Total weeks sampled (WIC); Initial PCV (PCI) and PCV at first screening (PCSR), which were taken before challenge; and some traits related to body weight (Traits 35–41), such as Initial Body Weight (WT1), which were thought to be primarily determined by loci affecting BW rather than by loci affecting trypanotolerance. For each trait, Table 6 shows the trait number, definition, trait group, acronym, direction of effect (H, L or NR), and mode of calculation.

**Table 6:** Traits analyzed and their definitions and mode of calculation

**No**^**a**^	**G**^**b**^	**T**^**c**^	**ACR**^**d**^	**Definition**	**Mode of calculation**
1	NR^k^	NR^k^	WIC	Total weeks sampled	Total number of weeks sampled over the entire challenge period, according to batch number
2	P^e^	L	STR	Total observed parasitemia detections	Total number of observed weekly positive parasitemia detections over entire challenge period.
3	P^e^	L	STRTV	Total observed *T. vivax* detections	Total number of observed positive *T. vivax* detections over entire challenge period.
4	P^e^	L	STRTC	Total observed *T. congolense* detections	Total number of observed positive *T congolense* detections over entire challenge period.
5	P^e^	L	TPS	Sum of parasitemic scores	Sum of all parasitemic scores over the entire challenge period
6	P^e^	L	MPAR	Mean parasitemic score	TPS/STR
7	P^e^	L	%PARD	Percentage of weeks animal parasitemic	100*STR/WIC
8	P^e^	L	NINF	Number of infection cycles	Total number of infection cycles, defined as number of new infections following initial exposure or treatment.
9	T^f^	L	NT	Number of treatments	Total number of treatments. If after first treatment PCV was still <18%, the animal was given a second treatment and this was counted as two treatments.
10	T^f^	L	NT1	Number of treatments when PCV <18%	Total No. of treatments given when PCV <18%
11	T^f^	H	NIT	Proportion of non-treated parasitemia detections	(STR-NT)/STR
12	T^f^	L	MNT	Mean treatments per week	NT/WIC
13	T^f^	L	MNTI	Mean treatments per week when PCV<18%	NT1/WIC
14	IC^1,g^	H	DF1	Days from exposure to first infection	No. of days from date animal is first exposed to tsetse challenge to date of first parasitemia detection
15	IC^1,g^	H	DT1	Days from first infection to first treatment for that infection	No. of days from date of first parasitemia detection to date of first treatment for that infection (in some cases a second treatment was needed to control the infection).
16	IC^1,g^	H	DC1A	Length of first infection cycle, counting from exposure to treatment	DF1+DT1; This is length of first infection cycle counting from exposure to treatment.
17	IC^1,g^	H	DF2	Days from first treatment to second infection	No. of days from date animal is first treated for first infection to date of second parasitemia detection (i.e., first parasitemia detection after final treatment for first infection).
18	IC^1,g^	H	DC1B	Length of first infection cycle counting from first infection	DT1+DF2; This is length of first infection cycle counting from first infection to second infection (after treatment for first infection).
19	IC^1,g^	H	DT2	Days from second infection to treatment for that infection	No of days from second parasitemia detection to first treatment for that infection.
20	IC^1,g^	H	DC2A	Length of second infection cycle, counting from first treatment for first infection to first treatment for second infection.	DF2+DT2
21	IC^1,g^	H	DC12	Total length of first two infection cycles counting from exposure through first treatment for second infection.	DF1+DT1+DF2+DT2 = DC1A+DC2A
22	PC^h^	NR^k^	PCI	Initial PCV	Mean PCV of animal before challenge
23	PC^h^	H	MPC	Mean PCV	Mean PCV of animal during the entire challenge period
24	PC^h^	H	MXPC	Maximum PCV	Maximum PCV of animal during the entire challenge period
25	PC^h^	H	MNPC	Minimum PCV	Minimum PCV of animal during the entire challenge period
26	PC^h^	H	PCSR	PCV at first screening	PCV of animal when first screened
27	PC^h^	H	PCF1	PCV at first infection	PCV of animal at first parasitemic detection
28	PC^h^	H	PCT1	PCV at the first treatment	PCV of animal when first treated after first detection of parasitemia
29	PC^h^	H	PCF2	PCV at the second infection	PCV of animal when infected for second time
30	PC^h^	H	PCT2	PCV at second treatment	PCV of animal when treated after second infection
31	PC^h^	H^2^	PCIF1	PCV change from before exposure to PCV at first infection	PCF1-PCI
32	PC^h^	H^2^	PCF1T1	PCV change from first infection to PCV at first treatment	PCT1-PCF1
33	PC^h^	H^2^	PCIT1	PCV change from before exposure to PCV at first treatment	PCT1-PCI
34	PC^h^	L,H^3^	PCT1F2	PCV change from first treatment to second infection	PCF2- PCT1
35	WT^i^	NR^k,4^	WTI	Initial body weight	Mean of body weight of animal before challenge (within a month before exposure)
36	WT^i^	NR^k,4^	MWT	Mean body weight	Mean of body weight of animal during entire challenge period
37	WT^i^	NR^k,4^	MXWT	Maximum body weight	Maximum body weight during the entire challenge period
38	WT^i^	NR^k,4^	MNWT	Minimum body weight	Minimum body weight during the entire challenge period
39	WT^i^	NR^k,4^	WTF1	Body weight at time of first infection	Body weight of animal at time of first parasitemia detection after initial exposure
40	WT^i^	NR^k,4^	WTT1	Body weight at first treatment	Body weight of animal at first treatment
41	WT^i^	NR^k,4^	WTF2	Body weight at second infection	Body weight of animal at second infection (i.e., at first parasitemia detection after first treatment)
42	WT^i^	NR^k,4^	WTE	Body weight at end of challenge period	Body weight of the animal in the last month of tsetse challenge period
43	WTC^j^	H^2^	WTIF1	Body weight change from initial body weight to first infection	WTF1 - WTI
44	WTC^j^	H^2^	WTIT1	Body weight change from initial body weight to weight at first treatment	WTT1 - WTI
45	WTC^j^	NR^k,4^	WTC	Mean Body weight change during challenge period	MWT - WTI
46	WTC^j^	NR^k,4^	WTC-W	Mean Body weight change per week during challenge	(WTC)/WIC
47	WTC^j^	H^2^	WTF1T1	Body weight change from first infection to first treatment	WTT1 – WTF1
48	WTC^j^	H^2^	WTT1F2	Body weight change from first treatment to second infection	WTF2 - WTT1
49	WTC^j^	H	WT1E	Body weight changefrom start to end of challenge period	WTE – WT1

^a^trait number; ^b^trait group; ^c^direction of effect on trypanotolerance, i.e., whether high (H) or low (L) trait value is associated with greater trypanotolerance; ^d^trait acronym. Trait groups: ^e^ parasitemia; ^f^treatments; ^g^infection cycles; ^h^PCV; ^i^body weight; ^j^body weight change; ^k^not primarily related to trypanotolerance.

^1^ First infection cycle counted from first detection of parasitemia after initial exposure to challenge to treatment of first infection; second infection cycle counted from first detection of parasitemia after treatment of first infection to treatment for second infection; third infection cycle counted from first detection of parasitemia after treatment for second infection, to treatment for third infection, and so on. If challenge period ended after infection but before treatment, this was counted as a complete infection cycle.

^2^Change generally negative. Therefore, a high algebraic value (i.e., small negative-value) means greater trypanotolerance, a low algebraic value (large negative-value) means lower trypanotolerance.

^3^ If the animal is susceptible there will be a large drop from infection to treatment, and hence a large bounce back after treatment. If the animal is tolerant, there will be a lower drop from infection to treatment (although still PCV <18%) and hence not so large a bounce back after treatment. Thus, a low value means greater trypanotolerance. However, it could also go the other way – following treatment a susceptible animal may not recover to the same extent as a tolerant animal.

^4^Traits 35 to 42, and 45, 46 are considered to be primarily affected by variation in body weight due to segregation of body weight QTL derived from the Boran and N’Dama, rather than by trypanotolerance QTL.

**Figure 2:**
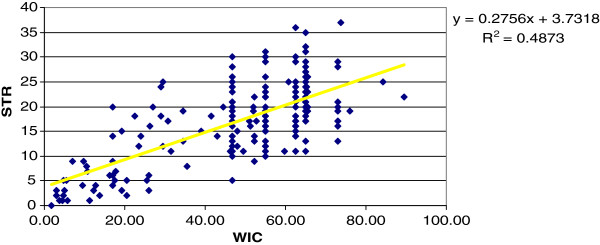
Scattergram of number of positive trypanosome cases detected (STR) against weeks in challenge (WIC).

Most of the defined traits were based on data obtained in the first two infection cycles, or on averages taken across all weeks sampled, and hence are more or less independent of the total number of weeks sampled (WIC). However, total observed parasitemia infections (STR), total number of infection cycles (NINF), total number of treatments (NT and NT1), and total weight change across the entire challenge period (WTC) can all be expected to show a linear relationship with WIC. See, e.g., Figure 2 showing scattergram of STR against WIC. A clear linear relationship is apparent (*R*^*2*^= 0.487 and *r* = 0.695). This is expected, as the longer the animals were exposed the more opportunity they had to be re-infected and thus present positive detection of trypanosomes.

On the assumption that for the time-related traits, the relation of trait value to WIC is approximately linear, they were standardized to the same challenge period by dividing by WIC, giving mean trait value per week. Thus, %PARD = 100*STR/WIC; MNT = NT/WIC; MNTI = NT1/WIC and WTCW = WTC/WIC.

### Phenotyping

All animals in the field were recorded weekly for packed cell volume (PCV) as a measure of anemia, and for parasitaemia score (PAR). PCV was determined by centrifugal techniques [19]. Parasitemia species scoring (whether *T. congolense* or *T. vivax*) was based on morphological features, while the concentration of parasites per ml of blood was estimated according to the parasitemia score [20] after examination of about 50 fields. Body weight (BW) measures of all animals were obtained in the morning every two weeks, on an electronic scale (Griffith Elder, UK) at ± 1 kg. When PCV was at 18% or less, animals were treated with a trypanocide (Berenil) by intramuscular injection at a dose of 7 mg/kg body weight. The number of treatments given to each animal in any given week (NT) was recorded as a fourth phenotype.

### Estimation of gender and batch effects

#### Fixed effect analysis of BCB animals for batch and gender effects

Batch and gender effects expressed as deviation from a reference group, and their statistical significance were estimated by a two-way ANOVA with batch and gender as main effects, using the GLM module of the SAS statistical package (SAS 9.1). For batch and gender effects, the males of Batch 1 served as the reference group, and batch and gender effects were estimated as deviation of respective batch or gender mean from the reference-group mean. This was done separately for each trait. Individual trait values were then corrected for gender and batch effects, using these estimates of gender and batch effects. For gender correction, only statistically significant (P≤0.05) gender-effect estimates were used. For batch correction, all batch effect estimates were used for trait correction, irrespective of statistical significance. This was done because the overall batch effects were highly significant for most traits, and were generally much more significant than the gender effects. Estimated mean values by batch for each trait were computed by adding respective batch effect to the reference mean value for that trait. In order to obtain a general picture of the overall behavior with respect to infections and treatments of the BCB animals across all batches, a parameter herein referred to as “MeanAll” (Table 1) was obtained for each trait, by calculating mean batch effect across all batches and adding this to the value of the reference group. Within-batch coefficients of variation (CV-within) were calculated for each trait, by computing standard deviation (SD) within each batch, and then mean SD pooled across batches; CV(within) = pooled SD/MeanAll. This within-batch CV was a measure of the ability of genetic and environmental factors varying within a given population and herd-year to affect trait value. In order to provide a similar parameter for the effects of between-batch environment on trait value, the between-batch coefficient of variation was calculated as CV (Batch) = SD (Between)/MeanAll, where SD (Between) is the standard deviation of batch effects across all batches.

#### Gender and Genetic-type effects on trypanotolerance

Female-gender effects as deviation from the male for the BCB animals across all six batches, were obtained from the two-way ANOVA as described above. Female-gender effects as a deviation from the male among the F1 in Batch 6 and among the KB in Batch 4, were separately analysed by batches, using a one-way ANOVA with gender as main effect. Genetic-type effects as a deviation from the BCB, were calculated for KB vs BCB in Batches 4 and 5, and for F1 vs BCB in Batch 6. Here too, the analyses were done separately by batches, using a one-way ANOVA with genetic type as main effect, after correcting for gender effects as obtained from the BCB, KB and F1 analyses.

The estimated effects between BCB and KB with their SE, were provided separately by ANOVA for Batch 4 and Batch 5 and then combined to a single estimate by calculating the simple unweighted mean of the two batch effects. SE of the combined effect was calculated as:

$$\;\begin{array}{cc} \text{SE}\;(\text{combined}\;\text{KB}\text{effect}) & =[({\text{SE}}_{2}\left(\text{Batch}\;4)\right)\\ & \;+\text{SE}(\text{Batch}5)]/4^{0.5} \end{array}$$

The effect of F1 as a deviation from KB was calculated as: Effect of F1 (as deviation from BC) - effect of KB (as deviation from BC), with

$$\;\begin{array}{cc} \text{SE} & =[{\text{SE}}^{2}\;\left(F1\;\text{to BCB}\;\text{effect}\right)\\ & \;+{\text{SE}}^{2}(\text{combined}\;\text{KB to BCB}\;\text{effect}{)]}^{0.5} \end{array}$$

Statistical significance of differences between genders and genetic types were tested using absolute z-values = |D|/SE (D). The corresponding *p*-value was obtained as twice the area to the right of z in the standard z-tables (two-tail test).

## Abbreviations

KARI: Kenya Agricultural research institute; KETRI: Kenya Trypanosomiasis research institute; TRC: Trypanosomiasis research centre; ILRI: International livestock research institute; QTL: Quantitative trait loci; ND: N’Dama; KB: Kenyan boran; BCB: Boran backcross; PCV: Packed cell volume; PC: PCV traits; IC: Infection cycle traits; TT: Treatment related traits; BW: Body weight; BWC: Body weight change traits; H: High trait value; L: Low trait value; NR: Not relevant; WIC: Total weeks sampled; PAR: Parastemic scores; STR: Total observed parasitemia detections; STRTV: Total observed *T. vivax* detections; STRTC: Total observed *T. congolense* detections; TPS: Sum of parasitemic scores; MPAR: Mean parasitemic score; %PARD: Percentage of weeks animal is parasitemic.; NINF: Number of infection cycles; NT: Number of treatments; NT1: Number of treatments when PCV <18%; NIT: Proportion of non-treated parasitemia detections; MNT: Mean treatments per week; MNTI: Mean treatments per week when PCV<18%; DF1: Days from exposure to first infection; DT1: Days from first infection to first treatment for that infection; DC1A: Length of first infection cycle, counting from exposure to treatment; DF2: Days from first treatment to second infection; DC1B: Length of first infection cycle counting from first infection; DT2: Days from second infection to treatment for that infection; DC2A: Length of second infection cycle, counting from first treatment for first infection to first treatme, t for second infection.; DC12: Total length of first two infection cycles counting from exposure through first treatment for second infection.; PCI: Initial PCV; MPC: Mean PCV; MXPC: Maximum PCV; MNPC: Minimum PCV; PCSR: PCV at first screening; PCF1: PCV at first infection; PCT1: PCV at the first treatment; PCF2: PCV at the second infection; PCT2: PCV at second treatment; PCIF1: PCV change from before exposure to PCV at first infection; PCF1T: PCV change from first infection to PCV at first treatment; PCIT1: PCV change from before exposure to PCV at first treatment; PCT1F2: PCV change from first treatment to second infection; WTI: Initial body weight; MWT: Mean body weight; MXWT: Maximum body weight; MNWT: Minimum body weight; WTF1: Body weight at time of first infection; WTT1: Body weight at first treatment; WTF2: Body weight at second infection; WTE: Body weight at end of challenge period; WTIF1: Body weight change from initial body weight to first infection; WTIT1: Body weight change from initial body weight to weight at first treatment; WTC: Mean Body weight change during challenge period; WTC-W: Mean Body weight change per week during challenge; WTF1T1: Body weight change from first infection to first treatment; WTT1F2: Body weight change from first treatment to second infection; WT1E: Body weight change from start to end of challenge period.

## Competing interests

None of the co-authors has competing interests.

## Authors’ contributions

CO carried out all genotyping, statistical and QTL mapping analyses, and wrote the article for BMC genetics, LM did the phenotyping, CK provided some guidance in the initial genotyping process (DNA extraction and quantification), JG, OH, AK and MS designed the study and provided project guidance and supervision. SK provided additional project guidance. MS participated in statistical analyses, and revised and edited the manuscript. All authors read and approved the final manuscript.
